# Preparing high purity white carbon black from rice husk

**DOI:** 10.1002/fsn3.1345

**Published:** 2019-12-18

**Authors:** Qunpeng Cheng, Chenxi Xu, Wenwen Huang, Chao Ma, Guozhi Fan, Juntao Yan, Zhang Jian, Yong Zhang, Guangsen Song

**Affiliations:** ^1^ School of Chemical and Environmental Engineering Wuhan Polytechnic University Wuhan Hubei China

**Keywords:** preparation, purity, rice husk, white carbon black

## Abstract

In this paper, rice husk (RH) was used as raw material to prepare white carbon black, and the key technological parameters of preparing white carbon black from RH were studied through single‐factor test, orthogonal experiment, and response surface analysis. Meanwhile, the characteristic of white carbon black was also analyzed. Through orthogonal experiment analysis, it was confirmed that the order of factors affecting the purity of white carbon black was calcination temperature > alkali treatment time > final pH > surfactant. Based on the response surface optimization analysis, the optimum parameters for preparation of white carbon black were as follows: calcination temperature 610°C, alkali treatment time of 2.3 hr, final pH of 10, CTMAB was used as the surfactant. Under this condition, the purity of silica prepared could be reached to 99.39%, and the particle size was uniform, spherical, and well dispersed, which satisfied the requirements of GB/T 34698‐2017 standard.

## Graphical abstract

The optimum parameters for preparation of white carbon black were as follows: calcination temperature 610°C, alkali treatment time of 2.3 hr, final pH of 10, and CTMAB was used as the surfactant. The purity of silica prepared could be reached to 99.39%, and it was satisfied the requirements of GB/T 34698‐2017 standard.

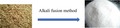

## 1 INTRODUCTION

Rice is the major food crop in China. During the process of multistage processing of rice, a great amount of rice husk (RH) was produced (Rafiee, Shahebrahimi, Feyzi, & Shaterzadeh, 2012). The rice production in China was approximately 228 million tons in 2011, which was resulted in approximately 45.6 million tons of RH (Xu, Sun, Guo, Zhang, & Dong, 2014). Usually, RH is burned to generate energy or stacked on farmland, which will cause environmental pollution and occupancy of landfill space (Shan et al., 2015). Therefore, the utilization of RH has been drawn widespread attentions. Due to high content of natural silica, producing white carbon black from RH is both economically beneficial and environmentally friendly (An, Guo, Bo, Zhu, & Wang, 2011).

White carbon black, as a kind of white, innocuous and amorphous powders, can be used in rubber, pesticide, feedstuff, catalyst carrier, and other fields (Jacobsen et al., 2010; Jiang, Wang, & Zhang, 2013; Shu, Shu, Zhi, & Li, 2010; Zhang, Tang, Xu, & He, 2011). Recently, white carbon black from RH is mainly prepared by chemical precipitation synthesis method in China because it is easy to control size, shape, and purity of the material (Fan, ; Xu et al., 2014; Yuvakkumar, Elango, Rajendran, & Kannan, 2014). Gao, Song, Yang, and Wu (2016) studied the preparation of white carbon black from water‐quenching blast furnace slag based on acid precipitation. The results showed that the quality of SiO_2_ in the product was increased from 84.43% to 91.32% after hydrochloric acid was used. Xu et al. (2014 prepared white carbon black from nano‐husk ash by analkali dissolving‐acid reaction method and investigated the effects of the pH and reaction time on the purity of the white carbon black. The results showed that the purity of white carbon black could reach to 98.48% when the NaOH solution with the RH ash was heated for 2 hr at 90°C and then finished its acid reaction at pH of 3 for 2 hr. Compared with acid precipitation method, alkali fusion method (involved dissolution of silica in NaOH solution followed by precipitation using acid solutions) was widely used. Shelke, Bhagade, and Mandavgane (2011) produced sodium silicate by reacting rice hull ash (RHA) with aqueous NaOH and precipitated silica from the sodium silicate by acidification. It was found that it was possible to recover over 90% of the silica contained in RHA. While white carbon black prepared by alkali fusion method usually contains Ca^2+^, Na^+^ (or K^+^), SO_4_^2−^ (or Cl^−^), and other metal impurities. Kalapathy, Proctor, and Shultz (2000a, 2000b) found that, before alkali treatment, washing RH ash with hydrochloric acid solution can effectively remove Ca^2+^ and metal impurities from RH, and washing silicate precipitation with distilled water can effectively remove Na^+^, K^+^, and Cl^−^ from white carbon black products. Later, Kalapathy, Proctor, and Shultz (2002) improved the method mentioned above. They prepared silica products with higher purity using oxalic acid and citric acid instead of hydrochloric acid.

The alkali fusion method is not only complicated, in which there are many factors affecting the quality and purity of white carbon black such as calcination temperature, final pH, and surfactant, but also is easily agglomerated. It is difficult to obtain high‐quality products with stable performance. Therefore, the precipitation method needs to be improved. Meanwhile, little researches were available on integrating process conditions to produce white carbon black. The aim of this study was to investigate the influence of calcination temperature, alkali treatment time, final pH, and surfactant on the purity of white carbon black to optimize the process of preparing white carbon black from RH. Meanwhile, the interaction of various factors on the influence of product purity was also investigated through the orthogonal experiment.

## 2 MATERIALS AND METHOD

### 2.1 Materials and pretreatment of RH

Rice husk was obtained from a rice mill in WuXue, Hubei province, China. It was first washed thoroughly with distilled water to remove impurities and then dried under 110°C for further use.

### 2.2 Preparation of white carbon black

**Figure 1:**
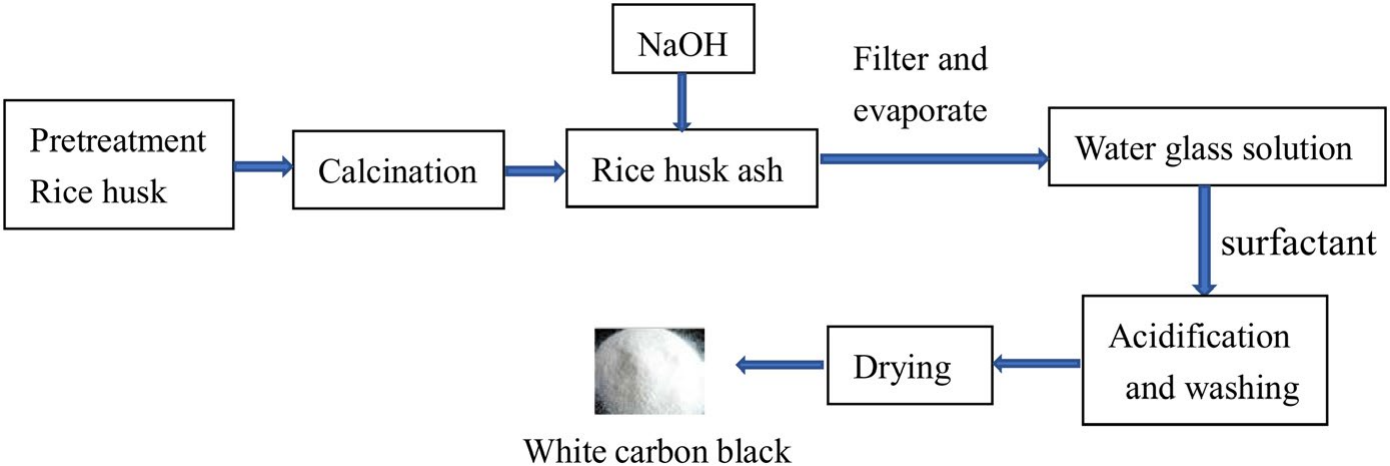
Diagram of whole experimental process

The basic process was shown in Figure 1, and the process was mainly divided into four steps.

1. The pretreated RHs were firstly calcined at 500, 600, 700, and 800°C respectively to obtain RH ash. The RH ash was crushed and evenly mixed for further use.
2. A total of 2.0 g of carbonized rice hulls with 40 ml 10% NaOH solution were put into 100 ml three‐flask and stirred by a magnetic stirring heater at 600 r/min under 120°C. The alkali treatment time was set as 1–4 hr to investigate the influence of alkali treatment time on the preparation process. After that, the mixture was washed and filtered. The residues were evaporated to 40 ml in a beaker at 80°C to obtain water glass solution.
3. To investigate the influence of surfactant on the content of SiO_2_ and particle morphology of white carbon black, 0.4 g PEG‐6000 (Polyethylene glycol 6000), SDS (sodium dodecyl sulfate), and CTMAB (cetyltrimethylammonium bromide) were added to water glass solution, respectively. Meanwhile, a blank control experiment was conducted. Then, the water glass solution was stirred on a magnetic stirring heater with a fixed reaction temperature 80°C at a speed of 600 r/min. 10% H_2_SO_4_ solution was intermittent added to the water glass solution until the white precipitate was slowly appeared. To investigate the influence of final pH on the particle size and purity of white carbon black, the final pH of reaction was regulated at 7–10.
4. After 2 hr reaction, the heating and stirring were stopped and the solutions were placed for 1 hr aging. Then, the precipitate was washed until the filtrate was neutral. Finally, the washed precipitate was drying in the drying oven and the product of white carbon black was obtained.

### 2.3 Analysis

The chemical composition of white carbon black was determined by X‐ray fluorescence (XRF). The morphology and the size of the samples were examined by S‐3000N scanning electron microscope (SEM). The spectra of the samples were recorded by using fourier transform infraredspectroscopy (FTIR, Nicolet 6700). Spectra were obtained from 4000 and 400 cm^−1^. The characteristic of white carbon black was evaluated according to GB/T 34698‐2017 standard (Rubber compounding ingredients—Determination of water‐soluble substance of silica, precipitated hydrated—Electrolytic conductivity method), China.

### 2.4 Orthogonal experiment

In order to verify the accuracy of single‐factor experimental results and to confirm the influence of various factors on the content of SiO_2_ in white carbon black, the orthogonal experiment was designed. Four factors including calcination temperature (A), alkali treatment time (B), final pH (C), and surfactant (D) were selected. Three levels were selected for each factor based on the yield and the purity of white carbon black in the single‐factor experiment. Orthogonal experiment was designed by using L_9_(3^4^) orthogonal table, which could be seen in Table 1.

**Table 1:** The design of Orthogonal experiment

	Calcination temperature (°C) A	Alkali treatment time (h) B	Surfactant C	Final pH D
1	600	2	None	8
2	700	3	CTMAB SDS	9
3	800	4	PEG‐6000	10

### 2.5 Response surface analysis

According to the previous experimental results of single‐factor and orthogonal experiment, calcination temperature (A), alkali treatment time (B), and final pH (C) were selected as the independent variable, the content of SiO_2_ in white carbon black (R) was selected as response values (dependent variable), and three factors and three levels of response surface experiments were designed using the Design‐Expert 10.0.4 software. The response surface analysis factors level code table was shown in Table 2.

**Table 2:** Design and results of response surface analysis

Item	A (°C)	B (h)	C	R (%)
1	700	3	9	96.928
2	700	3	9	96.758
3	700	4	8	95.159
4	700	3	9	97.728
5	700	2	8	95.186
6	600	2	9	98.253
7	800	2	9	97.203
8	700	3	9	96.728
9	600	3	10	98.406
10	800	4	9	94.768
11	800	3	8	95.305
12	700	2	10	98.333
13	600	3	8	97.830
14	600	4	9	96.438
15	800	3	10	97.821
16	700	4	10	97.106
17	700	3	9	96.086

## 3 RESULTS AND DISCUSSION

### 3.1 The effect of calcination temperature, alkali treatment time, final pH, and surfactant on the content of SiO_2_ in white carbon black

**Figure 2:**
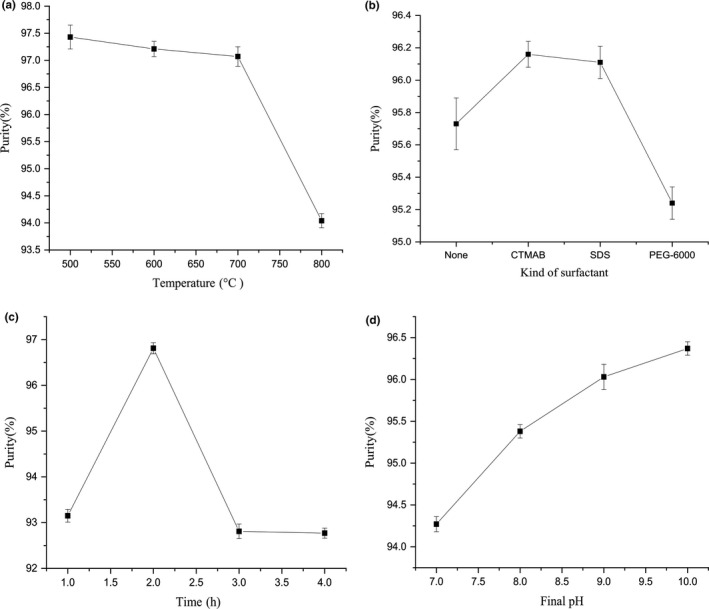
The effect of calcination temperature (a), alkali treatment time (b), surfactant (c), and final pH (d) on the purity of white carbon black

The effect of calcination temperature on the content of SiO_2_ in white carbon black was shown in Figure 2a. The calcination temperature was an important factor in the process of preparation of white carbon black, which was related to the properties of the final product like content of SiO_2_. The impurity content could be significantly reduced by thermal decomposition process. The metals in the RH were probably carried out from the volatiles during thermal decomposition (Liou, 2004). With the increase of the calcination temperature, the content of SiO_2_ was decreased. The highest content of SiO_2_ in white carbon black was appeared at 500°C. When the calcination temperature was in the range of 500–700°C, there was not obvious difference on the purity of white carbon black, while the purity of white carbon black was decreased rapidly when the temperature was over 700°C. The RH was burned completely, the content of porous carbon in RH ash was very low, impurities could not be adsorbed, and the purity of white carbon black products was relatively low. Meanwhile, when the temperature was above 700°C, amorphous silica was also transformed into the undesirable crystalline forms, such as crystobalite or tridymite, which was not benefit for synthesis into other products (Wattanasiriwech, Wattanasiriwech, & Svasti, 2010). However, due to the incomplete calcination of RH at 500°C, there was a lot of carbon left. Considering the energy consumption, purity and yield of white carbon black, the calcining temperature of RH was chosen at 600°C, which was in the range of 500–700°C taken by many researchers (Ping & Hsieh, 2012; Xu et al., 2014; Yuvakkumar et al., 2014).

The effect of alkali treatment time on the content of SiO_2_ in white carbon black was shown in Figure 2b. Water‐soluble sodium silicate was obtained by reacting with sodium hydroxide according to the Equation (1). When the alkali treatment time was less than 2 hr, the purity of white carbon black products was displayed as a rising trend and reached the maximum value at 2 hr. When the alkali treatment time was longer than 2 hr, the purity of white carbon black decreased. Longer alkali treatment time resulted in high concentration of silicate and promoting the growth and reunion of silicate nuclear, which would catch up more metal impurity and SO_4_^2−^during the acid process. Meanwhile, higher concentration of the silicate caused violent particle collisions and fast precipitation polymerization, which resulted in higher content of impurities in silica products. However, when the alkali treatment time was greater than 3 hr, impurities such as SiO_2_ and metals were completely dissolved, which resulted in not obvious variations on the purity of white carbon black. While when the alkali treatment time was less than 2 hr, the silica in RH was not completely dissolved, which lead to a lower production rate. Considering purity and yield of white carbon black, the alkali treatment time of RH was chosen at 2 hr.

(1) $${\text{SiO}}_{2}+2\text{NaOH}\to {\text{Na}}_{2}{\text{SiO}}_{3}+H_{2}O$$

**Figure 3:**
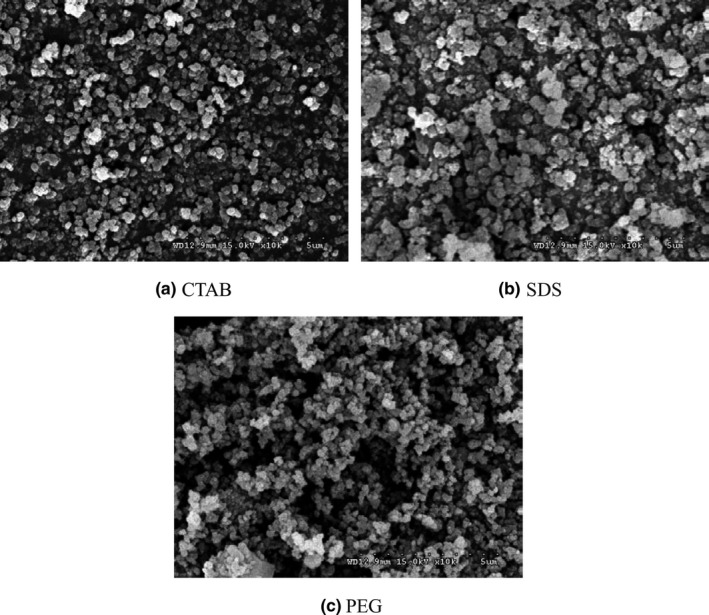
SEM micrographs of surfactants on the particle size and dispersion of white carbon black

The effect of surfactant on the content of SiO_2_ in white carbon black was shown in Figure 2c. It could be seen that the effect of surfactant on the purity of white carbon black was not significant. That was because the surfactant did not participate in the reaction, while surfactants mainly affect the particle size and dispersion of white carbon black. The addition of surfactant could reduce the surface tension of original grains and block the agglomeration of grains, which could greatly improve the quality of white carbon black (Li, Yu, & He, 2013). The SEM micrographs of surfactants on the particle size and dispersion of white carbon black were shown in Figure 3. The particles of white carbon black prepared by adding CTMAB were small and evenly distributed. While the particles of white carbon black prepared by adding SDS were not uniform in size and distribution, and the agglomeration phenomenon was serious. Uniform size, obvious agglomeration, and poor dispersion were observed on the white carbon black prepared by adding PEG‐6000. Hence, CTMAB was chosen as the surfactant in the later experiment.

The effect of final pH on the content of SiO_2_ in white carbon black was shown in Figure 2d. Silica was gradually precipitated to form a gel by sulfuric acid solution at pH 7 (Equation (2)).The purity of white carbon black was increased gradually with the rising of the final pH. Lower final pH needs more sulfuric acid, which caused higher concentration of H^+^ and faster precipitation polymerization speed. Meanwhile, the impurities were easier to be trapped and packaged resulting in lower purity of white carbon black. While when the final pH was more than 11, the yield of white carbon black was decreased. Tzong‐Horng Liou investigated effect of gelation pH and found that silica yields were increased with the rising of pH, with a maximum yield occurring at around pH 7. While when the pH was 11, these gels were almost dissolved in water (Liou & Yang, 2011). Considering the yield and purity of white carbon black, the final pH value was better to controlled at 10.

(2) $${\text{Na}}_{2}{\text{SiO}}_{3}+H_{2}{\text{SO}}_{4}\to {\text{SiO}}_{2}\cdot H_{2}O+{\text{Na}}_{2}{\text{SO}}_{4}$$

### 3.2 Results of orthogonal experiment

The results of orthogonal experiment were shown in Table 3. The range analysis showed that the orders of factors affecting the purity of white carbon black were A > B>C > D, that is, the calcination temperature > alkali treatment time > final pH > surfactants. Meanwhile, the results of variance analysis showed that calcination temperature, alkali treatment time, and final pH made significant influence on the purity of white carbon black, while the type of surfactant had not significant effect on white carbon black purity.

**Table 3:** Code and level of factors

Item	Code of factors	Horizontal coded values	Level value
Calcination temperature (°C)	A	−1	600
		0	700
		+1	800
Alkali treatment time (h)	B	−1	2
		0	3
		+1	4
Final pH	C	−1	8
		0	9
		+1	10

According to the results of orthogonal experimental, the best technological condition for preparing white carbon black from RHs was A1B1C3, that is, the calcination temperature was 600°C, the alkali treatment time was 2 hr, the final pH was 10, and the purity was the highest, which was consistent with those results of single‐factor experiment.

### 3.3 Results of response surface analysis

**Figure 4:**
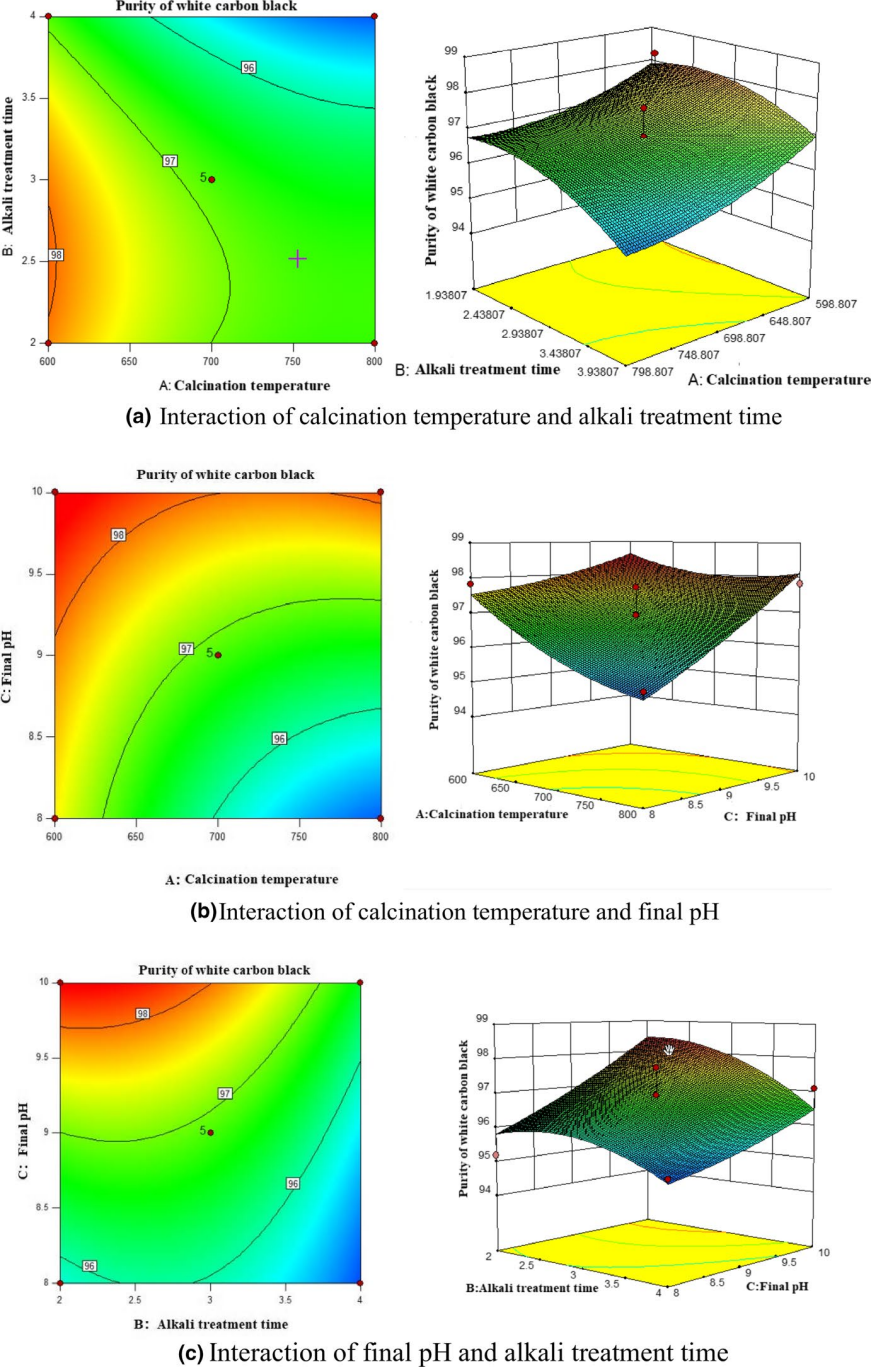
The interaction of calcination temperature, alkali treatment time, and final pH on purity of white carbon black

The interactions among calcination temperature**,** alkali treatment time and final pH were shown in Figure 4a–c. When C was fixed, the content of SiO_2_ in white carbon black was decreased with the rising of A and was first increased and then decreased with the rising of B (Figure 4a).When B was fixed, the purity of white carbon black was decreased with the rising of A and was increased with the rising of C (Figure 4b).When A was fixed, the purity of white carbon black was first increased and then decreased with B, but was increased with the rising of C (Figure 4c). This was consistent with the results of single‐factor experiment.

The results of response surface analysis were displayed in Table 4 and Table 5. It could be seen that the model was significant (*p* = .0224 < .05), and the misfit value of the model was not significant (*p* > .1), indicating a high fit of the model. Meanwhile, the influence of single factor in the model A (calcination temperature)**,** B (alkali treatment time), and C (final pH) was significant, while the interactions among AB, AC, and BC were not significant, which indicated that the interactions among calcination temperature, alkali treatment time, and final pH did not make significant influence on the purity of white carbon black.

**Table 4:** The results of orthogonal experiment

Number	A	B	C	D	Purity (%)
1	1	1	1	1	97.498
2	1	2	2	2	97.055
3	1	3	3	3	97.469
4	2	1	2	3	98.269
5	2	2	3	1	97.042
6	2	3	1	2	96.134
7	3	1	3	2	94.985
8	3	2	1	3	92.541
9	3	3	2	1	92.949
K_1_	292.022	290.752	286.173	287.489	
K_2_	291.445	286.638	288.273	288.174	
K_3_	280.475	286.552	289.496	288.279	
k_1_	97.34067	96.91733	95.391	95.82967	
k_2_	97.14833	95.546	96.091	96.058	
k_3_	93.49167	95.51733	96.49867	96.093	
Range	3.849	1.4	1.107667	0.263333	
SS	28.223	3.841377	1.883118	0.122706	
Total	863.942				
*p* = *T* ^2^/*n*	82932.86				

Analysis of variance differences	SS	*df*	MS	F	Significance	
A	28.223	2	14.1115	230.0059	**	Highly significant
B	3.841377	2	1.920688	31.30565	**	Highly significant
C	1.883118	2	0.941559	15.34664	**	Highly significant
D	0.122706	2	0.061353			
Error	0.122706	2	0.061353			
Δe	0.245411	4	0.061353			
*F*_0.01_(2, 4) = 18		*F*_0.05_(2, 4) = 6.94				

**Table 5:** ANOVA results of response surface analysis

Sources of variation	Sum of squares	Degree of Freedom	Mean‐square value	*F* value	Probability >*F*	Significance
Model	19.55	9	2.17	5.03	0.0224	+
A	4.25	1	4.25	9.83	0.0165	+
B	3.79	1	3.79	8.76	0.0211	+
C	8.38	1	8.38	19.38	0.0031	+
AB	0.096	1	0.096	0.22	0.6516	—
AC	0.94	1	0.94	2.18	0.1836	—
BC	0.36	1	0.36	0.83	0.3918	—
A^2^	0.54	1	0.54	1.24	0.3017	—
B^2^	1.22	1	1.22	2.81	0.1375	—
C^2^	0.080	1	0.080	0.18	0.6803	—
Residual	3.03	7	0.43			
Lack of fit	1.64	3	0.55	1.58	0.3260	—
Pure Error	1.38	4	0.35			
Total difference	22.58	16				

Abbreviations: —, not significant; +, significant.

The process parameters of preparing white carbon black from RH were optimized and analyzed by Design‐expert 10.0.4 software: the calcination temperature was 612°C, the alkali treatment time was 2.29 hr, the final pH was 9.99, and the maximum purity of white carbon black could be reached to 99.94%.

### 3.4 Characteristic of white carbon black

**Figure 5:**
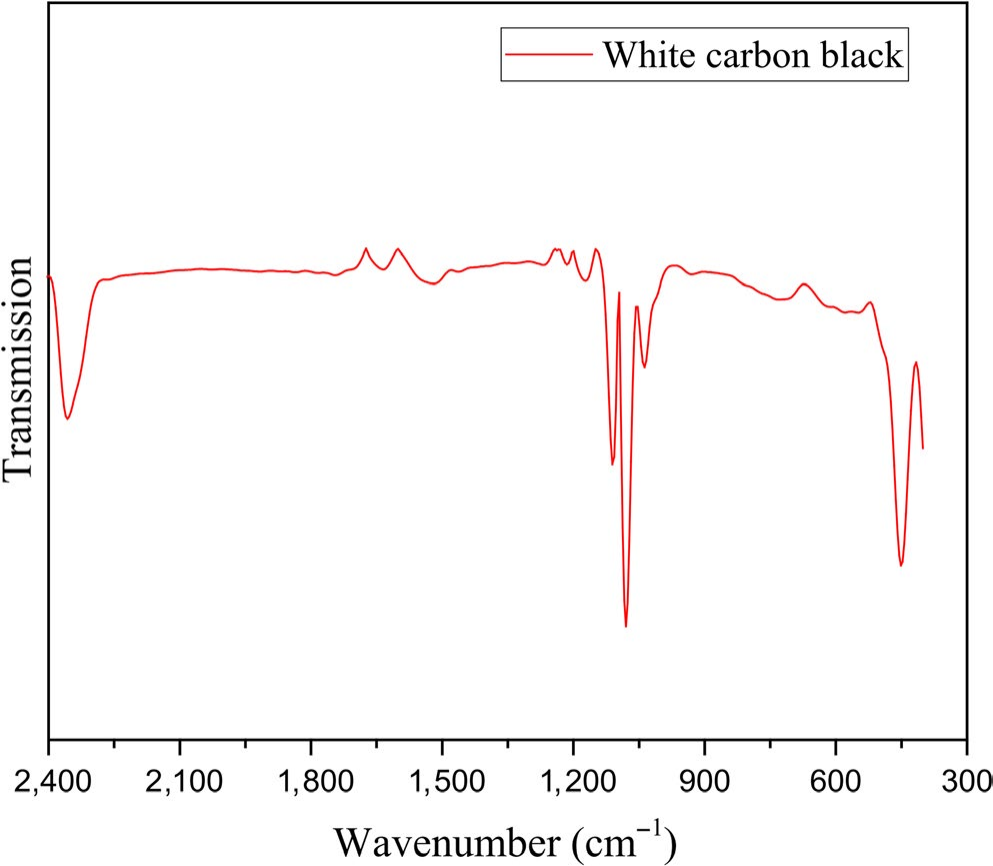
FTIR spectra of white carbon black

FTIR spectra of white carbon black were shown in Figure 5. The absorption peaks in 1109.77, 967.66, and 417.04 cm^−1^ were the characteristic absorption peaks of SiO_2_, in which 1109.77 and 417.04 cm^−1^ were stretching vibration absorption peak of Si‐O‐Si asymmetric and symmetric stretching modes (Gao et al., 2016; Khan, Saha, Sultana, & Ahmed, 2017); 967.66 cm^−1^ was corresponded to the bending vibration absorption peak of Si‐O‐(H, H_2_O) (Zhi & Guo, 2013). It indicated that the silica prepared in this experiment was amorphous hydrated silica with high purity and very low impurity content.

**Figure 6:**
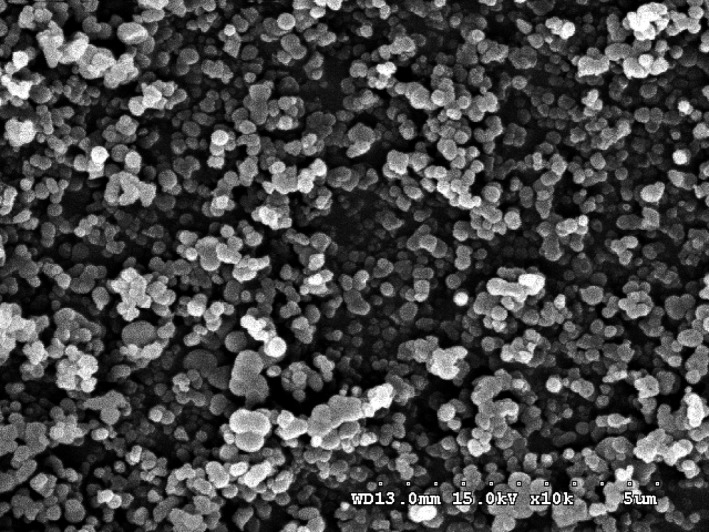
SEM micrographs of white carbon black prepared under optimized condition

Table 6 showed the characteristic of white carbon black. It indicated that the prepared white carbon black products met the index requirements of GB/T 34698‐2017 standard. Meanwhile, the SEM micrographs of white carbon black prepared under optimized condition were shown in Figure 6, which was small and evenly distributed. The silica content of all samples was higher than 90%, which satisfied the standards and market quality requirements.

**Table 6:** The characteristic of white carbon black

Item	Standard	Product
SiO_2_ (%, d)	≥90	99.39
Residue on sieve (%, d)	≤0.5	0.1
Loss on ignition (%, d)	≤7.0	1.7
pH	5.0–8.0	6.4
Cu (mg/kg)	≤10	None
Mn (mg/kg)	≤40	13.2
Fe (mg/kg)	≤500	86.0

Abbreviation: d, dry.

## 4 CONCLUSION

The purity of white carbon black was decreased with the rising of calcination temperature, while was increased with the rising of final pH. With the rising of alkali treatment time, it was first increased and then decreased. The order of factors affecting the purity of white carbon black was as follows: calcination temperature > alkali treatment time > final pH > surfactant. Meanwhile, the influence of interaction among those three factors on the purity of white carbon black was not significant. The optimal process parameters for preparation white carbon black from RH: calcination temperature was 610°C, alkali treatment time was 2.3 hr, the final pH was 10, CTMAB was used as the surfactant, and it could obtain a purity of over 99% white carbon black.

## CONFLICT OF INTEREST

The authors declare that we do not have any conflict of interest.

## ETHICAL REVIEW

This study does not involve any human or animal testing.
